# P-777. Assessment of Epidemiologic Case Definitions for Disseminated Nontuberculous Mycobacterial Disease in Patients Without AIDS

**DOI:** 10.1093/ofid/ofae631.971

**Published:** 2025-01-29

**Authors:** Kelly A Jackson, Rebecca Byram, Christopher A Czaja, Helen Johnston, Kelsey T Orten, Ruth Lynfield, Nathan Centurion, Laura Tourdot, Paula Snippes Vagnone, Ghinwa Dumyati, christopher J Myers, Shannon C O’Brien, Adel M Mansour, D Rebecca Prevots, Kevin L Winthrop, Nadege Charles Toney, Shelley Magill, Isaac See

**Affiliations:** U.S. Centers for Disease Control and Prevention, Atlanta, Georgia; Division of Healthcare Quality Promotion, Centers for Disease Control and Prevention (CDC); Chenega Enterprise Systems and Solutions, Atlanta, Georgia; Colorado Department of Public Health and Environment, Denver, Colorado; Colorado Department of Public Health, Denver, Colorado; Colorado Department of Public Health and Environment, Denver, Colorado; Minnesota Department of Health, St. Paul, MN; Minnesota Department of Health, St. Paul, MN; Minnesota Department of Health, St. Paul, MN; Minnesota Department of Health Laboratory, St. Paul, MN; New York Emerging Infections Program and University of Rochester Medical Center, Rochester, New York; University of Rochester, Rochester, New York; Oregon Health Authority, Portland, Oregon; Oregon Health Authority, Portland, Oregon; National Institute of Allergy and Infectious Diseases, Bethesda, MD; OHSU-PSU School of Public Health, Portland, Oregon; Division of Healthcare Quality Promotion, Centers for Disease Control and Prevention, Atlanta, Georgia; Centers for Disease Control and Prevention, Atlanta, GA; U.S. Centers for Disease Control and Prevention, Atlanta, Georgia

## Abstract

**Background:**

Disseminated *Mycobacterium avium* complex (MAC) infection rates have declined in persons with HIV; some studies report increasing numbers of disseminated nontuberculous mycobacteria (NTM) infections in other groups of immunocompromised persons. However, no consensus definition exists for disseminated NTM in these populations. We compared characteristics of disseminated NTM in persons without AIDS using different epidemiologic definitions.

**Table 1.**
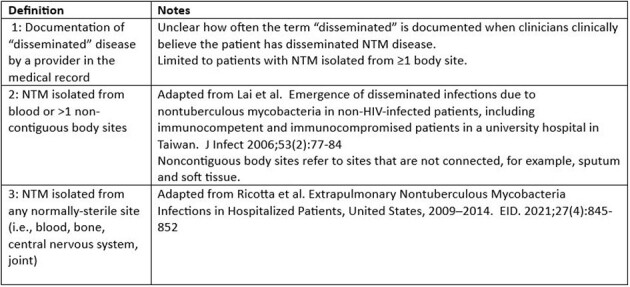
Disseminated nontuberculous mycobacteria definitions..

**Methods:**

CDC’s Emerging Infections Program conducted active, laboratory- and population-based NTM surveillance in 4 states (11 counties) during 2021–2022 to identify patients with NTM in clinical specimens, excluding stool or rectal swabs. Clinical and laboratory data were collected via medical record review. Disseminated NTM definitions are shown in Table 1. We determined the numbers of patients without AIDS who met each definition. Among those meeting each definition we calculated the percentages of patients with: 1) selected immunocompromising conditions; 2) MAC infection; and 3) documented clinical diagnosis of any NTM infection (i.e., NTM not likely to represent contamination or colonization).

**Figure 1.**
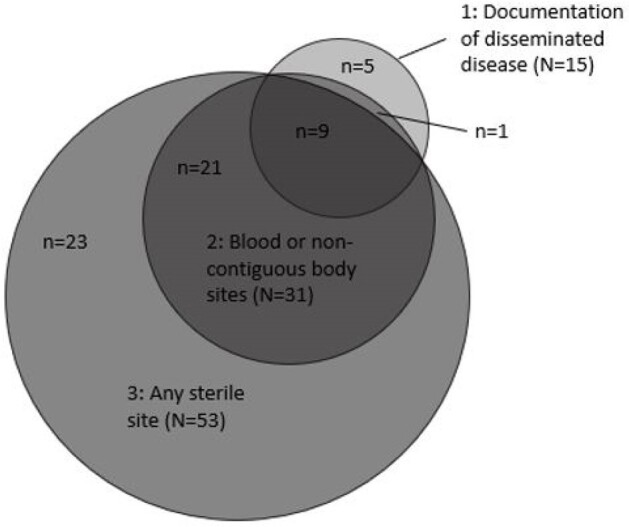
Number of patients meeting each disseminated nontuberculous mycobacteria definition, Emerging Infection Program, 2021–2022 (N=59).

**Results:**

Among 877 patients with NTM and without AIDS, 15 patients (1.7%) had a documented disseminated NTM clinical diagnosis (definition 1); 59 (6.7%) met ≥1 of the 3 definitions. Although more patients met definition 2 (31, 3.5%) or definition 3 (53, 6.0%) than definition 1, definition 2 only captured 10/15 (66.7%) patients with a documented disseminated NTM clinical diagnosis; definition 3 captured only 9/15 (60.0%) (Figure 1). The proportion of patients with malignancy, immunosuppressive medication, or transplant differed by definition as did the proportion infected with MAC. Seven of 31 patients meeting definition 2 (22.6%) and 14/53 (26.4%) patients meeting definition 3 did not have a clinician documented diagnosis of any NTM infection in the medical record (Figure 2).

**Figure 2.**
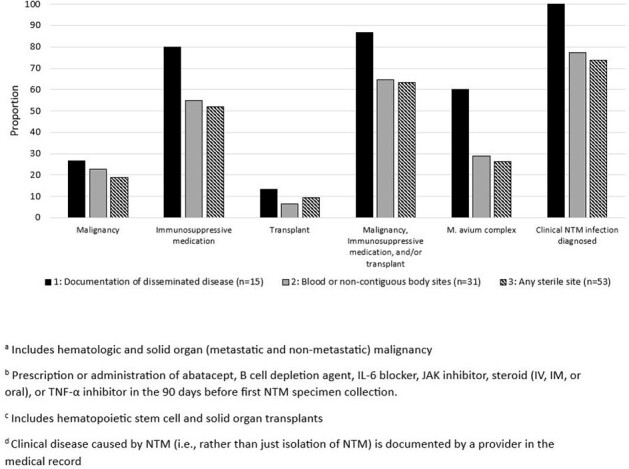
Proportion of patients with malignancy(a), immunosuppressive medication(b), transplant(c), isolation of M. avium complex, or clinical diagnosis of any type of nontuberculous mycobacteria infection(d), stratified by disseminated NTM definition, Emerging Infections Program, 2021–2022.

**Conclusion:**

Each disseminated NTM definition identified a different group of patients, limiting their usefulness and comparability. Development of a standard epidemiologic definition for disseminated NTM in persons without AIDS will enable better understanding of disease impact in susceptible populations.

**Disclosures:**

**Kevin L. Winthrop, MD, MPH**, AN2: Advisor/Consultant|AN2: Grant/Research Support|Insmed: Advisor/Consultant|Insmed: Grant/Research Support|Mannkind: Advisor/Consultant|Mannkind: Grant/Research Support|Paratek: Advisor/Consultant|Paratek: Grant/Research Support|Renovion: Advisor/Consultant|Renovion: Grant/Research Support|Spero: Advisor/Consultant|Spero: Grant/Research Support

